# The *BCL2*-938 C > A promoter polymorphism is associated with risk group classification in children with acute lymphoblastic leukemia

**DOI:** 10.1186/1471-2407-13-452

**Published:** 2013-10-02

**Authors:** Annette Künkele, Anja Grosse-Lordemann, Alexander Schramm, Angelika Eggert, Johannes H Schulte, Hagen S Bachmann

**Affiliations:** 1 Department of Pediatric Hematology-Oncology, University Hospital Essen, Hufelandstrasse 55, 45147, Essen, Germany; 2 Institute of Pharmacogenetics, University Hospital Essen, Hufelandstrasse 55, 45147, Essen, Germany

**Keywords:** Pediatric ALL, *BCL2*-938C > A, Risk stratification

## Abstract

**Background:**

Acute lymphoblastic leukemia (ALL) is the most common pediatric cancer. While current treatment regimens achieve almost 80% overall survival, long-term side effects of chemotherapeutic agents can be severe. The functional *BCL2*-938C > A promoter polymorphism is known to influence the balance between survival and apoptosis of malignant hematolymphoid cells. We investigated its usefulness as a marker for treatment stratification for children with ALL.

**Methods:**

We analyzed DNA from 182 children suffering from ALL in this study to determine genotypes of the−938 C > A polymorphism by “slow-down” PCR.

**Results:**

ALL patients with the *BCL2*-938CC genotype had an approximately 3-fold higher risk of belonging to a high-risk group. Within the high-risk group, 50% of *BCL2*-938CC patients were classified as high-risk due to poor prednisone response whereas only 33% of patients with AC and AA genotypes were classified as high-risk for the same reason.

**Conclusions:**

Our results suggest that *BCL2*-938C > A genotyping may be beneficial for therapy response prediction in ALL patients, and warrant examination in a larger cohort to validate its usefulness for treatment stratification of pediatric ALL patients.

## Background

Leukemia is the most common pediatric cancer and the most common cause of disease-related death in childhood. Acute lymphoblastic leukemia (ALL) accounts for 80% of pediatric leukemias [1]. The disease is characterized by uncontrolled proliferation of lymphoid progenitors in the bone marrow with consequent accumulation of malignant lymphoblasts in both bone marrow and peripheral blood. Current treatment regimens result in approximately 80% overall survival (OS) in children with ALL. However, the chemotherapeutic agents used target both malignant and non-malignant cells, and the side effects can be severe, especially for high-risk patients, who receive the most aggressive therapy [1-3]. Particularly, long-term side effects are of great interest in pediatric oncology, since these patients are treated very early in life, with many years following their disease. The identification of additional markers which would allow improved risk stratification and more individually tailored therapy regimens is highly desirable, even to lower risk of adverse long-term side effects. For the time being the most important outcome predictors are genetic subsets, initial white blood cell count, age at diagnosis, and early treatment response measured by minimal residual disease (MRD) markers [4]. Although many genetic abnormalities are associated with clinical outcome, only few of them are routinely used for treatment stratification. These include the presence of trisomy 4, 10 or 17 or the Philadelphia chromosome. Occasionally the significance of a given prognostic factor may change with advances in treatment. For example, the outcome for pediatric ALL patients with the Philadelphia chromosome has improved substantially since tyrosine kinase inhibitors were included in the treatment regimen [5]. Finding markers for risk stratification can improve risk group assignment and consequently improve treatment efficacy by applying as much therapy as needed to cure the patient but avoiding over-treatment, which can increase long-term adverse side effects. Bcl-2 was initially identified as an anti-apoptotic regulatory protein [6], but it also serves as an inhibitor of proliferation [7]. The functional implication of Bcl-2 in tumorigenesis is ambiguous, as the anti-apoptotic effect may be oncogenic while the anti-proliferative effect appears tumor-suppressive. Which of the dual functions predominates seems to be tissue specific. This may explain why the prognostic implication of Bcl-2 expression depends on cancer type. For example, its expression is related to decreased survival in chronic lymphocytic leukemia and prostate cancer [8,9], whereas increased Bcl-2 expression is associated with favorable outcome in colorectal and breast cancer [10,11]. Bcl-2 may play a crucial role in the survival of hematolymphoid cells, where Bcl-2 inhibits apoptosis by preventing cytochrome c release from the mitochondria, thereby blocking caspase activation [12]. *BCL2* has two promoters with different functional properties. Transcription is driven by the P1 promoter, while the P2 promoter acts as a negative regulatory element [13,14]. *Cis* alterations have been shown to increase the activity of the P2 promoter, suppressing Bcl-2 expression [14]. The functional−938 C > A promoter polymorphism is located in the inhibitory P2 promoter region [15]. C and A alleles of this polymorphism differentially bind transcription factors and affect promoter activity. The C allele shows altered binding of transcription factors, increased activity of inhibitory promoter P2 and therefore decreased expression of Bcl-2. In patients with lymph node-negative breast cancer, oropharyngeal squamous cell carcinoma, chronic lymphocytic leukemia, glioblastoma multiforme and prostate cancer the A allele has been associated with higher Bcl-2 expression levels and the−938 C > A polymorphism has been associated with survival of patients suffering from these cancers [16-20]. The present study investigates genotypes of the *BCL2*−938 C > A promoter polymorphism as potential markers for risk and treatment stratification in children with ALL.

## Methods

### Patient cohort

Research followed the tenets of the Declaration of Helsinki, and was approved by the institutional review board of Children’s University Hospital Essen. The study comprised blood samples from 182 patients diagnosed with ALL and treated between January 1987 and December 2010 in the Department of Pediatric Hematology-Oncology of the University Hospital Essen according to the clinical trial protocols ALL-BFM 1990, 1995 and 2000, Interfant, EsPhALL and CoALL for which informed consent was obtained. Data were retrospectively evaluated from medical records.

### Determination of *BCL2*−938C > A genotypes

To determine the *BCL2*−938 genotypes, DNA was isolated from peripheral blood by using a commercially available kit according to the manufacturer’s instructions (QIAamp, Qiagen, Hilden, Germany). Genotypes of the−938 C > A polymorphism were determined by slowdown PCR [21]. Previously described primers were used for the region of interest: forward primer 5′-CAGCAGCTTTTCGGAAAATG-3′ and biotinylated reverse primer 5′-TATCCACGGGACCGCTTCTTCAC-3′ [18]. The 134 bp PCR products were analyzed by pyrosequencing using the primer 5′-TCCCCGGCTCCTTCATCGTC-3′ on the PSQ96 system according to the manufacturer’s instructions (Biotage, Uppsala, Sweden), and as previously described [18]. Results were analyzed using PSQ96 SNP software (Biotage, Uppsala, Sweden). Appropriate negative and positive controls for each genotype were included. Sequencing accuracy was verified by re-genotyping 60 samples using pyrosequencing and 10 randomly selected samples by Sanger sequencing (100% concordance with results from slowdown PCR). Slowdown PCR and pyrosequencing assays have been extensively used and validated for polymorphism genotyping in previous studies [16-18].

### Statistical analysis

This study used a hypothesis-driven retrospective candidate gene approach to investigate the *BCL2*−938C > A polymorphism. Kaplan-Meier analyses and the log-rank test were used to retrospectively evaluate the relationship between risk group, *BCL2* genotype and outcome from the date of primary diagnosis to the end of follow-up. Multiple Cox regression analyses were performed to assess the joint impact of *BCL2*−938C > A genotype, patient gender and age with or without risk group classification on the clinical outcome. Student’s t-test was used for genotype-dependent comparison of continuous variables. Contingency tables and Pearson’s chi-squared test were used to compare categorical variables dependent on *BCL2* genotypes. AA and AC genotypes have been combined as one group to allow for odds ratio analysis and linear testing versus risk groups. Deviation from the Hardy-Weinberg equilibrium was assessed using the Hardy-Weinberg equilibrium public domain software by S. Rodriguez *et al*. [22] (http://www.oege.org/software/hwe-mr-calc.shtml). Differences with p < 0.05 were regarded as significant. Statistical analyses were conducted using SPSS 17.0 (IBM, Ehningen, Germany), or GraphPad Prism 4.0 (GraphPad Software Inc., La Jolla, CA, USA).

## Results

We retrospectively genotyped a series of 182 children diagnosed and treated for ALL in the Children’s University Hospital Essen for the *BCL2*−938C > A polymorphism, for which the patient characteristics are presented in Table 1. Mean age of patients at diagnosis was 6.5 (range 0.1-17.7) years. The median follow-up for patients without fatal events was 5.2 years and the median age at recruiting for follow-up was 11.7 (range 1.2-30.2) years. Event-free survival (EFS) and 5-year OS of the entire patient cohort was 71% and 88.5%, respectively. From the initial cohort, 29 patients died from relapse (n = 16), bronchiolitis obliterans and respiratory insufficiency (n = 1), multiple organ toxicity (n = 6), renal failure (n = 1), infection (n = 4) or due to unknown causes (n = 1). The initial leukocyte count was between 0 and 20 000 leukocytes/nl for 112 patients, between 20 000 and 100 000 leukocytes/nl for 49 patients, above 100 000 leukocytes/nl for 19 patients and data was unavailable for 2 patients. Central nervous system involvement was detected in 6 patients. Molecular genetic abnormalities are summarized in Table 1. Patient immunophenotyping confirmed common ALL in 126 patients, early pre-B ALL in 28 patients, pre-B ALL in 7 patients, pre-T ALL in 2 patients, an intermediate T-ALL in 12 patients and a mature T-ALL in 1 patient. Immunohistological diagnosis of the ALL subtype was not possible in 6 patients. In this cohort, 160 patients responded well and 14 responded poorly to prednisone, whereas data were unavailable for 8 patients about prednisone response. The cohort analyzed here was treated under several different treatment protocols, including the ALL BFM 2000 protocol (n = 126), the ALL-BFM 1995 protocol (n = 26), the ALL-BFM 1990 protocol (n = 14), the Interfant protocol (n = 6), the EsPhALL protocol (n = 6), the CoALL protocol (n = 4). Of the initial cohort, 45 patients relapsed, with the median time from diagnosis to relapse being 2.4 (range 0.2-5.5) years and 63 patients underwent hematopoietic stem cell transplantation, with the median time between initial diagnosis of ALL and hematopoietic stem cell transplantation being 2.1 (range 0.3-9.1) years. Of the patients who underwent hematopoietic stem cell transplantation 25 relapsed following therapy.

**Table 1:** Clinico-pathological characteristics at primary diagnosis and genotype distribution

	**All**	***BCL2*−938 genotype**		***P *value**
		**AC / AA**	**CC**	
n (%)	182	141 (77.5)	41 (22.5)	
Mean age (years ± SD)	6.5 ± 4.6	6.3 ± 4.5	7.1 ± 4.6	0.298
Gender (male/female)	111/71	83/58	28/13	0.276
Relapse				
no	137	105 (76.7)	32 (23.3)	0.640
yes	45	36 (80.0)	9 (20.0)	
HSCT				
no	119	97 (81.5)	22 (18.5)	0.073
yes	63	44 (69.9)	19 (30.1)	
Relapse after HSCT (n = 63)				
no	40	26 (65.0)	14 (35.0)	0.270
yes	23	18 (78.3)	5 (21.7)	
Risk Group Classification				
standard	67	59 (88.1)	8 (11.9)	
medium	72	53 (73.6)	19 (26.4)	0.008
high	43	29 (67.4)	14 (32.6)	
Prednisone response (n = 174)				
good	160	129 (80.6)	31 (19.4)	0.148
poor	14	9 (64.3)	5 (35.7)	
Molecular genetics (n = 176)				
no aberration	121	94 (77.7)	27 (22.3)	0.627
BCR/ABL fusion	13	9 (69.2)	4 (30.8)	
TEL/AML rearrangement	35	28 (80.0)	7 (20.0)	
MLL/AF4 rearrangement	3	3	0	
TCF3/PBX1 rearrangement	1	1	0	
chromosomal 9 deletion	1	1	0	
hyperdiploidity	2	2	0	

HSCT, Hematopoietic stem cell transplantation.

**Figure 1:**
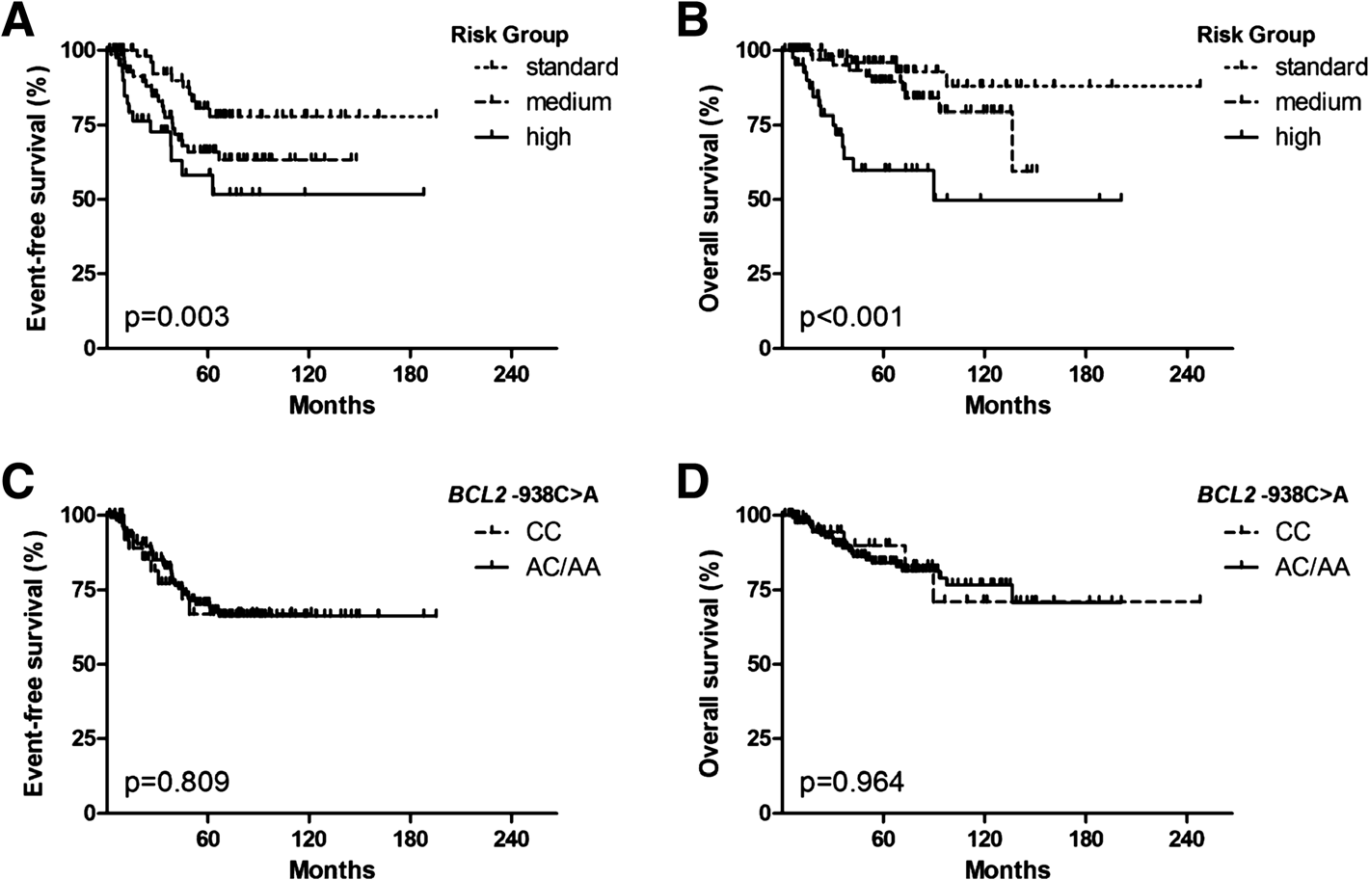
**Risk assessment of ALL patients treated within several different trial treatment regimens correlated with patient outcome.** Kaplan-Meier curves are shown for the 182 pediatric ALL patients assessed retrospectively in this study. Patients were treated within ALL BFM 2000, ALL-BFM 1995, ALL-BFM 1990, Interfant, EsPhALL, CoALL and CoALL/BFM. **A-B**. Patients with a standard risk (SR) ALL have a significant better EFS and OS than those with a medium (MR) or high risk (HR) ALL. **C-D**. There is no significant difference concerning EFS or OS between combined AC/AA genotype and CC genotype of the *BCL2*−938C > A polymorphism.

The genotype distribution of the *BCL2*−938C > A polymorphism was representative of healthy Caucasians. It consisted of n = 41 CC, n = 85 AC and n = 56 AA, which is in line with the Hardy-Weinberg equilibrium. The frequency of the C allele within this cohort (0.46) did not significantly differ from published data from healthy Caucasian cohorts [16,18]. The patient genotype of the *BCL2*−938C > A polymorphism was not correlated with patient outcome or with clinical risk markers, including prednisone response or identified molecular genetic anomalies. No difference concerning OS or EFS between good or poor prednisone responders or between the identified molecular genetic anomalies was detected (Data not shown). As expected, risk groups were significantly correlated with OS. Patients categorized in standard-risk groups according to the respective protocol had a significantly better OS than the patients categorized in medium-or high-risk groups (Figure 1A and B). Carrier status of either the CC or the AC/AA genotype of the *BCL2*−938 C > A polymorphism did not significantly correlate with either patient EFS or OS (Figure 1C and D). We could show that the percentage of patients with a CC genotype increases linearly from the standard–(11.9%), through the medium–(26.4%) and high-risk groups (32.6%, p = 0.008; Table 1). A child with a CC genotype has a significantly higher risk to belong to the medium-or high-risk groups instead of the standard-risk group (OR = 2.64 [95%CI 1.07-6.54); p = 0.031 and OR = 3.56 [95%CI 1.34-9.45]; p = 0.008, Table 2).

**Table 2:** ***BCL2*−938C > A genotype and ALL risk groups**

	***BCL2*−938 genotype**			
Risk group	CC	AC / AA	Odds Ratio (95% CI)	*P* value
standard	8 (11.9)	59 (88.1)	1.00	
medium	19 (26.4)	53 (73.6)	2.64 (1.07-6.54)	0.031
high	14 (32.6)	29 (67.4)	3.56 (1.34-9.45)	0.008

CI, Confidence Interval.

To further investigate the discrepancy between risk group assignment and outcome, we performed multivariate regression analyses and exploratory analyses of the high-risk group. Multiple Cox regression analyses revealed that *BCL2*−938C > A genotype remained non-significant for EFS and OS even after correction for different covariables and especially after correction for the risk group classification (Additional file 1: Table S1). Poor therapy response as well as genomic aberration of tumor cells leads to classification of patients as high risk. Interestingly, 77% of patients with the CC genotype were classified as high risk due to poor response to therapy, whereas only 55% of patients with the AC/AA genotype. Poor therapy response is defined by poor response to prednisone or by detection of MRD at certain time points during therapy. Remarkably, the putative difference in therapy response seems to be related to prednisone. In high risk patients with available prednisone response data (n = 37) 50% of the children with the CC genotype were classified as high-risk because of poor prednisone response, while only 33% of the AC/AA patients were classified as high-risk due to poor prednisone response (Table 3). In conclusion, we here identified the *BCL2*-938 CC genotype as a novel risk factor predisposing childhood ALL patients to a higher treatment risk group, although overall survival was similar to patients in all risk groups.

**Table 3:** **High Risk patients with available prednisone response data and *BCL2*-938C > A genotype**

	**All**	***BCL2*-938 genotype**	
		**AC / AA**	**CC**
n (%)	37	27 (73.0)	10 (27.0)
High risk due to poor prednisone response			
no	23 (62.2)	18 (66.7)	5 (50.0)
yes	14 (37.8)	9 (33.3)	5 (50.0)

## Discussion

Treatment of ALL involves some of the most complex chemotherapy combinations and treatment schedules used in oncology. In most patients, induction chemotherapy is initially used to reduce the burden of lymphoblasts in the bone marrow and restore normal hematopoietic function. Consolidation therapy is then used with the intention of clearing all drug-resistant leukemia cells that have survived induction therapy from the blood and bone marrow and to prevent minimal residual disease. Maintenance chemotherapy consists of two to three years of low-dose antineoplastic drugs designed to prevent leukemia relapse during the crucial few years after remission, induction and consolidation. The significant improvements in survival for children with ALL during the past two decades can be attributed to both favorable disease biology and the success of carefully designed, risk-adapted treatment. Risk stratification evolved during these decades to incorporate response to initial treatment, cytogenetics and other biological characteristics of the disease. Measurement of minimal residual disease is now used to improve risk stratification and to allocate patients to hematopoetic stem cell transplantation in many pediatric clinical trials.

In this study, we examined the *BCL2*−938 C > A polymorphism as possible marker for risk stratification and predicting outcome in pediatric ALL patients. The long follow-up time of more than 60 months allowed the evaluation of OS and EFS. Both were comparable to that reported for other cohorts of pediatric ALL patients with similar follow-up periods [23-25]. Since pediatric cancers are relatively rare, only those treatment centers seeing a large proportion of children with cancer are able to recruit sufficient numbers of patients for retrospective analyses of long-term follow-up events, and this takes a long time to be accomplished. The patients analyzed in our study were treated according to seven different protocols. The variety in the protocols stem from changes of trial protocols made during the long recruitment times and age-adjusted treatment regimens. This permanent change and development can complicate analyses and comparability of analyses, in addition to the small numbers of patients in subgroups with specific molecular genetics backgrounds or ALL immunophenotyping. Some limitations exist for the interpretation of the data in this study, since patients were non-uniformly treated and some results were detected in (exploratory) subgroup analysis, in which some subgroups contained small number of patients. The effect of the *BCL2*−938 C > A promoter polymorphism should be assessed in a larger independent cohort of, optimally, uniformly treated patients.

The patient genotype of the *BCL2*−938C > A polymorphism was significantly correlated with risk stratification, but not with outcome or the clinical risk markers examined. As expected, risk groups were significantly correlated with OS and EFS, so that patients belonging to the low-risk groups had a significantly better outcome than the patients belonging to the medium-or high-risk groups [26,27]. This supports that patients belonging to a higher risk group require a more intensive treatment regimen. Since more intensive treatment regimens can cause severe side effects and/or long-term adverse affects, additional markers to improve selection of the best treatment regimen have the power to reduce both short-and long-term adverse patient effects. Bcl-2 is known to be a possible indicator for therapy response during induction chemotherapy treatment of adult patients with ALL [28], and Bcl-2 inhibition in combination with methadone increases the induction of apoptosis in pediatric ALL cells [29]. In our study, the−938 C > A *BCL2* promoter polymorphism did not correlate with OS or EFS and is on its own not predictive of outcome of pediatric ALL patients, but we detected an influence of the *BCL2*−938 C > A polymorphism on risk stratification. In our cohort, the risk of a child with a CC genotype to be in a specific risk group increased significantly and linearly from the standard-to high-risk groups, and vice versa, decreased linearly for patients with either AA or AC genotypes. In an exploratory analysis, poor therapy response, and especially response to prednisone was the main reason for classification of *BCL2*−938 CC patients into the high-risk group, although their overall outcome was not worse than the outcome of patients belonging to standard-or medium-risk groups. Since it is known that prednisone treatment decreases Bcl-2 expression and ALL patients are routinely treated with prednisone, this polymorphism should be assessed in ALL patients [30]. Furthermore, Bcl-2 is an interesting drug target with different inhibitors under evaluation and the polymorphism identified here would be an interesting candidate to investigate its putative biomarker properties [31,32].

## Conclusion

Our results suggest that *BCL2*−938 C > A genotyping may be beneficial for predicting therapy response in ALL patients. This polymorphism should be examined in a larger independent (prospective) cohort to verify the connection between risk and genotype and validate its usefulness for treatment stratification of pediatric ALL patients with regard to its possible interaction with prednisone response.

## Competing interests

The authors declare that they have no competing interests.

## Authors’ contributions

AK has made substantial contributions to acquisition, analysis and interpretation of data, design of the study and writing the manuscript. AG was involved in acquisition and analysis of data. AS and AE have been involved in drafting the manuscript and revising it critically for important intellectual content. JHS has made contributions to conception and design of the study and performed the statistical analysis. HSB has made substantial contribution in analysis and interpretation of data, design of the study and revising the manuscript. All authors read and approved the final manuscript.

## Pre-publication history

The pre-publication history for this paper can be accessed here:

http://www.biomedcentral.com/1471-2407/13/452/prepub

## Supplementary Material

Additional file 1: Table S1 Cox regression analyses. Click here for file
