# Supplementary material for: The BCL2-938 C > A promoter polymorphism is associated with risk group classification in children with acute lymphoblastic leukemia
Source: BMC Cancer. 2013 Oct 2;13:452. doi: 10.1186/1471-2407-13-452 (PMC3850706; doi:10.1186/1471-2407-13-452)
Supplement: Additional file 1: Table S1 — Cox regression analyses. [file 1471-2407-13-452-S1.docx]

| **Supplemental Table 1. Cox regression analyses** | | | | | | | | |
| --- | --- | --- | --- | --- | --- | --- | --- | --- |
|  |  |  |  | Multivariate | | | | |
|  | Univariate | |  | Model 1 | |  | Model 2 | |
| Covariable | HR (95% CI) | *P* value |  | HR (95% CI) | *P* value |  | HR (95% CI) | *P* value |
| *Event-free Survival* | | | | | | | | |
| *BCL2* -938 |  |  |  |  |  |  |  |  |
| AC / AA | 1 |  |  | 1 |  |  | 1 |  |
| CC | 1.094 (0.53-2.28) | 0.809 |  | 1.084 (0.51-2.30) | 0.833 |  | 0.864 (0.40-1.86) | 0.708 |
| Gender |  |  |  |  |  |  |  |  |
| male | 1 |  |  | 1 |  |  | 1 |  |
| female | 0.858 (0.47-1.58) | 0.623 |  | 0.864 (0.47-1.60) | 0.640 |  | 0.807 (0.44-1.49) | 0.493 |
| Age | 1.000 (0.99-1.01) | 0.950 |  | 1.000 (0.99-1.01) | 0.930 |  | 0.998 (0.99-1.01) | 0.529 |
| Risk group |  |  |  |  |  |  |  |  |
| standard | 1 |  |  | - |  |  | 1 |  |
| medium | 2.028 (0.96-4.31) | 0.066 |  | - |  |  | 2.140 (1.00-4.58) | 0.050 |
| high | 3.290 (1.46-7.44) | 0.004 |  | - |  |  | 3.651 (1.57-8.51) | 0.003 |
|  |  |  |  |  |  |  |  |  |
| *Overall Survival* | | | | | | | | |
| *BCL2* -938 |  |  |  |  |  |  |  |  |
| AC / AA | 1 |  |  | 1 |  |  | 1 |  |
| CC | 0.978 (0.37-2.58) | 0.964 |  | 0.932 (0.35-2.50) | 0.889 |  | 0.717 (0.26-1.95) | 0.514 |
| Gender |  |  |  |  |  |  |  |  |
| male | 1 |  |  | 1 |  |  | 1 |  |
| female | 1.884 (0.89-3.98) | 0.097 |  | 1.841 (0.87-3.92) | 0.113 |  | 1.810 (0.85-3.86) | 0.125 |
| Age | 1.005 (0.99-1.01) | 0.138 |  | 1.005 (0.99-1.01) | 0.153 |  | 1.002 (0.99-1.01) | 0.656 |
| Risk group |  |  |  |  |  |  |  |  |
| standard | 1 |  |  | - |  |  | 1 |  |
| medium | 2.298 (0.72-7.35) | 0.161 |  | - |  |  | 2.274 (0.71-7.40) | 0.167 |
| high | 8.353 (2.73-25.58) | <0.001 |  | - |  |  | 8.268 (2.62-26.11) | <0.001 |
| HR, Hazard Ratio; CI, Confidence Interval | | | | | | | | |
|  | | | | | | | | |
